# Transmembrane Topology and Signal Peptide Prediction Using Dynamic Bayesian Networks

**DOI:** 10.1371/journal.pcbi.1000213

**Published:** 2008-11-07

**Authors:** Sheila M. Reynolds, Lukas Käll, Michael E. Riffle, Jeff A. Bilmes, William Stafford Noble

**Affiliations:** 1 Department of Electrical Engineering, University of Washington, Seattle, Washington, United States of America; 2 Department of Genome Sciences, University of Washington, Seattle, Washington, United States of America; 3 Department of Biochemistry, University of Washington, Seattle, Washington, United States of America; 4 Department of Computer Science and Engineering, University of Washington, Seattle, Washington, United States of America; Columbia University, United States of America

## Abstract

Hidden Markov models (HMMs) have been successfully applied to the tasks of transmembrane protein topology prediction and signal peptide prediction. In this paper we expand upon this work by making use of the more powerful class of dynamic Bayesian networks (DBNs). Our model, *Philius*, is inspired by a previously published HMM, Phobius, and combines a signal peptide submodel with a transmembrane submodel. We introduce a two-stage DBN decoder that combines the power of posterior decoding with the grammar constraints of Viterbi-style decoding. Philius also provides protein type, segment, and topology confidence metrics to aid in the interpretation of the predictions. We report a relative improvement of 13% over Phobius in full-topology prediction accuracy on transmembrane proteins, and a sensitivity and specificity of 0.96 in detecting signal peptides. We also show that our confidence metrics correlate well with the observed precision. In addition, we have made predictions on all 6.3 million proteins in the Yeast Resource Center (YRC) database. This large-scale study provides an overall picture of the relative numbers of proteins that include a signal-peptide and/or one or more transmembrane segments as well as a valuable resource for the scientific community. All DBNs are implemented using the Graphical Models Toolkit. Source code for the models described here is available at http://noble.gs.washington.edu/proj/philius. A Philius Web server is available at http://www.yeastrc.org/philius, and the predictions on the YRC database are available at http://www.yeastrc.org/pdr.

## Author Summary

Transmembrane proteins control the flow of information and substances into and out of the cell and are involved in a broad range of biological processes. Their interfacing role makes them rewarding drug targets, and it is estimated that more than 50% of recently launched drugs target membrane proteins. However, experimentally determining the three-dimensional structure of a transmembrane protein is still a difficult task, and few of the currently known tertiary structures are of transmembrane proteins despite the fact that as many as one quarter of the proteins in a given organism are transmembrane proteins. Computational methods for predicting the basic topology of a transmembrane protein are therefore of great interest, and these methods must be able to distinguish between mature, membrane-spanning proteins and proteins that, when first synthesized, contain an N-terminal membrane-spanning signal peptide. In this work, we present *Philius*, a new computational approach that outperforms previous methods in simultaneously detecting signal peptides and correctly predicting the topology of transmembrane proteins. Philius also supplies a set of confidence scores with each prediction. A Philius Web server is available to the public as well as precomputed predictions for over six million proteins in the Yeast Resource Center database.

## Introduction

The structure of a protein determines its function. Knowledge of the structure can therefore be used to guide the design of drugs, to improve the interpretation of other information such as the locations of mutations, and to identify remote protein homologs.

Indirect methods such as X-ray crystallography and nuclear magnetic resonance spectroscopy are required to determine the tertiary structure of a protein. Membrane proteins are essential to a variety of processes including small-molecule transport and signaling, and are of significant biological interest. However, they are not easily amenable to existing crystallization methods, and even though some of the most difficult problems in this area have been overcome in recent years, the number of known tertiary structures of membrane structures remains very low. Computational methods that can accurately predict the basic topology of transmembrane proteins from easily available information therefore continue to be of great interest. To be most valuable, a predicted topology include not only the locations of the membrane-spanning segments, but should also correctly localize the N- and C-termini relative to the membrane.

Many proteins include a short N-terminal signal peptide that initially directs the post-translational transport of the protein across the membrane and is subsequently cleaved off after transport. A signal peptide includes a strongly hydrophobic segment which is not a part of the mature protein but is often misclassified as a membrane-spanning portion of a transmembrane protein. Conversely, a transmembrane protein with a membrane-spanning segment near the N-terminus is often misclassified as having a signal peptide. Therefore, signal peptide prediction and transmembrane topology prediction should be performed simultaneously, rather than being treated as two separate tasks.

Membrane proteins are classically divided into two structural classes: those which traverse the membrane using an *α*-helical bundle, such as bacteriorhodopsin, and those which use a *β*-barrel, such as porin. The *β*-barrel motif is found only in a small fraction of all membrane proteins (e.g., in the outer membrane of Gram negative bacteria and in the mitochondrial membrane). Lately, some attention has been given to some irregular structures such as re-entrant loops and random coil regions. In this work, however, we focus on the *α*-helical class, both because most membrane proteins fall into this class, and because they constitute most of the known 3D structures.

The two most common machine learning approaches applied to the prediction of both signal peptides and the topology of transmembrane proteins are hidden Markov models (HMM) and artificial neural networks (ANN), while some predictors use a combination of these two approaches. HMMs are particularly well suited to sequence labeling tasks, and task-specific prior knowledge can be encoded into the structure of the HMM, while ANNs can learn to make classification decisions based on hundreds of inputs.

The first HMM-based transmembrane protein topology predictors were introduced ten years ago: TMHMM [1] and HMMTOP [2]. Both of these predictors define a set of structural classes which capture the variation in amino acid composition of different portions of the membrane protein. For example, the membrane-spanning helix is known to be highly hydrophobic, and cytoplasmic loops generally contain more positively charged amino acids than non-cytoplasmic loops (the so-called positive-inside rule). During training the HMM learns a set of emission distributions, one for each of the structural classes. TMHMM is trained using a two-pass discriminative training approach followed by decoding using the one-best algorithm [3]. HMMTOP introduced the hypothesis that the difference between the amino acid distributions in the various structural classes is the main driving force in determining the final protein topology, and that therefore the most likely topology is the one that maximizes this difference for a given protein. HMMTOP [4] was also the first to allow constrained decoding to incorporate additional evidence regarding the localization of one or more positions within the protein sequence. The presence of a signal peptide within a given protein has also been successfully predicted using both HMMs [5] and ANNs [6].

As mentioned above, the confusion between signal peptides and transmembrane segments is one of the largest sources of error both for conventional transmembrane topology predictors and signal peptide predictors [7],[8]. Motivated by this difficulty, the HMM Phobius [9] was designed to combine the signal peptide model of SignalP-HMM [5] with the transmembrane topology model of TMHMM [1]. The authors showed that including a signal peptide sub-model improves overall accuracy in detecting and differentiating proteins with signal peptides and proteins with transmembrane segments.

In this work, we introduce *Philius*, a combined transmembrane topology and signal peptide predictor that extends Phobius by exploiting the power of dynamic Bayesian networks (DBN). The application of DBNs to this task provides several advantages, specifically: (a) a new two-stage decoding procedure, (b) a new way of expressing non-geometric duration distributions, and (c) a new approach to expressing label uncertainty during training. Philius is inspired by Phobius and tackles the problem of discriminating among four basic types of proteins: globular (G), globular with a signal peptide (SP+G), transmembrane (TM), and transmembrane with a signal peptide (SP+TM). Philius also predicts the location of the signal peptide cleavage site and the complete topology for membrane proteins.

We report state-of-the-art results on the discrimination task and improvements over Phobius on the topology prediction task. We also introduce a set of confidence measures at three different levels: at the level of protein type, at the level of the individual topology segment (e.g., inside, membrane, outside), and at the level of the full topology. Confidence measures for topology predictions were introduced by Melén et al. [10], and we expand upon this work with these three types of scores that correlate well with the observed precision.

Finally, based on the Philius predictions on the entire Yeast Resource Center [11] protein database, we provide an overview of the relative percentages of different types of proteins in different organisms as well as the composition of the class of membrane proteins.

### Background

Transmembrane protein topology prediction can be stated as a supervised learning problem over amino acid sequences. The training set consists of pairs of sequences of the form (***o***,***s***) where ***o*** = *o*_1_,…,*o_n_* is the sequence of amino acids for a protein of known topology, and ***s*** = *s*_1_,…,*s_n_* is the corresponding sequence of labels. The *o_i_* are drawn from the alphabet of 20 amino acids 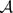, and the *s_i_* are drawn from the alphabet of topology labels, 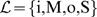, corresponding respectively to cytoplasmic (“inside”) loops, membrane-spanning segments, non-cytoplasmic (“outside”) loops, and signal peptides. After training, a learned model with parameters Θ takes as input a single amino acid test sequence ***o*** and seeks to predict the ‘best’ corresponding label sequence ***s*** * (with no unknowns).

We solve this problem using a DBN, which we call Philius. Before describing the details of our model, we first review HMMs and explain how they are a simple form of DBN. The generality of the DBN framework provides significantly expanded flexibility relative to HMMs, as described in [12]. A recently published primer [13] provides an introduction to probabilistic inference using Bayesian networks for a variety of applications in computational biology.

### Hidden Markov Models

HMMs are conceptually simple and yet also almost unlimited in their flexibility [14]. An HMM is a generative model in which an observed sequence is generated according to an underlying but unknown sequence of states. More precisely, an HMM is a joint probability distribution over a set of 2*N* variables: the *N* observations **o**, and the *N* hidden states, **s**. The HMM assumes that the joint distribution over these 2*N* variables can be factorized as follows:
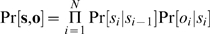
(1)where **s** = {*s*_1_,…,*s_N_*}, **o** = {*o*_1_,…,*o_N_*}, 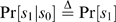, and where *i* represents position along the observed sequence. An HMM is often used to compute the probability distribution over the observations Pr[**o**] by summing (or marginalizing) over all possible hidden state sequences **s** in the above joint distribution. An HMM might also be used as a means to infer a most probable sequence of states from an input sequence of observations. The factorization property of an HMM makes these sorts of computations (collectively called *statistical inference*) based on an HMM tractable, and has been one of the keys to their widespread success.

The two conditional relationships that define an HMM are generally constant with respect to the position *i*. An HMM such as this is referred to as a *time-homogeneous* model (since the parameters are homogeneous with respect to time). This time-homogeneity allows the HMM to represent sequences of states and observations of arbitrary length *N* with a fixed and finite number of parameters. Most HMMs and dynamic Bayesian networks are time-homogeneous.

It is perhaps most common in the literature to represent an HMM using a *state transition graph* in which each node is a state in the model, and directed edges between pairs of nodes show the allowed (non-zero probability) transitions between states. Such a graph shows only the allowable state transitions–nothing in this graph describes the observation distributions Pr[*o_i_*|*s_i_*] nor is anything stated about the HMM joint distribution and the factorization properties mentioned in Equation 1.

### HMMs as Bayesian Networks

**Figure 1:**
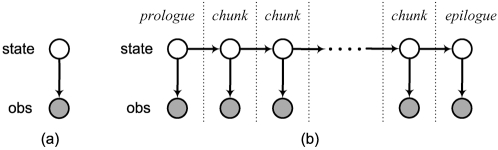
Hidden Markov model. (a) BN with two variables which constitutes the basic (single frame) template for an HMM, and (b) A DBN representation of an HMM obtained by concatenating a variable number of the BN frames and connecting successive state variables.

In many applications and publications using HMMs, the HMM state transition diagram may be the only descriptive graphic provided. In our research, we often use in addition a quite different graphical description of an HMM, one that depicts a very different set of HMM properties. As mentioned above, Equation 1 makes explicit the factorization properties of an HMM, and these properties allow for efficient inference on the HMM. We can use a type of graph known as a Bayesian network (BN) to visually and precisely convey this set of properties, as is done in Figure 1. Figure 1a shows the “static” relationship between a state variable and the associated observation at a single point *i* corresponding to the factor Pr[*o_i_*|*s_i_*] in Equation 1. Figure 1b shows the graph for the expanded HMM corresponding to Equation 1, which includes a node for each state and observation variable for all time-points *i* = 1,…,*N*. This figure makes clear the dynamic aspect of the model, i.e., Pr[*s_i_*|*s_i_*_−1_] and Pr[*o_i_*|*s_i_*] for all *i*. A *Bayesian network* (BN) is one type of graphical model in which edges are directed, and in which directed cycles are not allowed [15].

A *frame* (often also referred to as a *slice* or *time-slice*) in an HMM corresponds to one vertical section, corresponding to a single time point *i*. For example, in order to model a protein of length *N*, we could use an HMM that consists of *N* frames, where each amino acid has its own local copy of the basic HMM template. In an HMM, this slice contains only two random variables. We refer to the first and last frames as the *prologue* and *epilogue* of the model respectively, and to each frame in between as a *chunk*. In order to create an HMM of length *N*, the chunk is replicated *N*−2 times, a process sometimes referred to as *unrolling*. The prologue and epilogue often differ slightly from the chunk, allowing for distinct modeling at the extreme ends of the sequence. In the BN representation, we follow the convention that shaded nodes represent *observations* (also collectively referred to as the *evidence*), while unshaded nodes represent *hidden* variables. The chain of hidden variables is where the HMM gets its name–there is a presumed underlying set of hidden variables that form a (first order) Markov chain.

The BN representation of an HMM illustrates the minimum factorization properties required of a joint probability distributions that fits the model. More generally, the use of the term *graphical model* [16], implies that there is a graph (a set of nodes and edges) in which nodes correspond to random variables, and edges encode in a mathematically precise way the set of conditional independence (or factorization) properties of any probability distribution over those random variables which can be represented by the graph.

### Dynamic Bayesian Networks

*Dynamic Bayesian networks* (DBNs) are BNs that extend over time (or some other dimension such as genomic or protein sequence position). DBNs are strict generalizations of both HMMs and BNs and are constructed in much the same way: by concatenating identical (except possibly the first and last) copies of a “static” BN and linking the adjacent BN copies together in some consistent way. The same advantage of being able to model sequences of essentially unbounded length using a finite number of parameters that gives the HMM much of its power carries over naturally to the DBN. In fact, any HMM is an instance of a DBN—Figure 1a shows the static BN which when repeated over and over gives us the DBN description of an HMM in Figure 1b. The converse, that any DBN is an instance of an HMM, is however not true.

#### More variables can mean fewer free parameters

**Figure 2:**
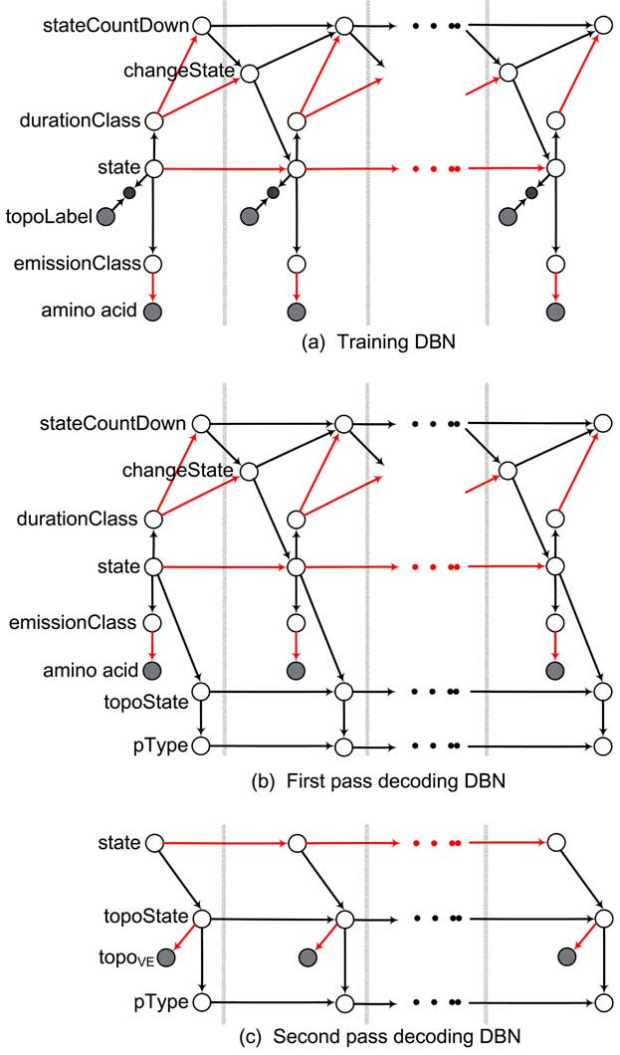
Philius training and decoding graphical models. (a) Training DBN: only the amino acid and the topoLabel are observed in each frame. The topoLabel is used to constrain the hidden state using an observed child node. The color of the edge between two nodes indicates the type of relationship: black is deterministic, and red is random. (b) First stage decoding DBN: the topoState is hidden and dependent on the state and the previous topoState, and specifies the behavior of pType, an additional hidden variable. (c) Second stage decoding DBN: the observed amino acid node and the duration modeling nodes have been removed, and Pr[*topoState_i_*] is defined by the posterior probabilities computed in the first stage using the virtual evidence node topo*_VE_*.

DBNs gain flexibility over HMMs because, in a DBN, the repeated static BN is not limited to be a network with two variables as in Figure 1a. For example, Figure 2 shows three DBNs where each repeated frame consists of multiple random variables. The relationship between the variables is expressed by a graph, and like any BN the graph conveys factorization properties of any joint distribution that is to be represented by the DBN. As with the HMM, it is the factorization properties of a DBN that (may) allow for efficient inference.

The flexibility to define more than two variables in each frame, as well as more than one connection between adjacent frames has several advantages. While it is sometimes possible to bundle all the variables in a DBN frame into a single HMM “super-variable”, such an HMM super-variable loses the factorization and explicit relationships between variables that can be expressed in a DBN. This loss of factorization can lead to substantial computational costs for an equivalent HMM as compared to a DBN, as well as a dramatically increased number of free parameters.

In any machine learning setting, it is important to control the model complexity, in particular when the amount of training data is limited. Tying of parameters is one way to control the number of free parameters and hence model complexity. Parameter-tying is implicit in all time-homogeneous DBNs (including HMMs) because parameters are tied across time. The flexibility to specify a larger number of variables within each frame of the DBN brings with it the ability to also tie parameters within a single time slice.

#### Constrained inference

Any factorization of the joint probability distribution of a set of random variables, which can be expressed as a graphical model, implies a set of constraints. The topology of the traditional HMM is one way of describing and constraining the relationships between the states and the observations. It has become common practice to impose additional constraints on HMMs, typically during decoding, by implementing customized versions of common algorithms [4],[10]. The DBN framework permits these types of constraints to be expressed directly within the graph topology [17], without requiring any changes to the underlying inference algorithms.

A variety of constraints based on prior knowledge can be built into a DBN and can be used both during training and decoding to preclude certain combinations of variable assignments by specifying that these combinations have zero probability. In fact, training on labeled examples can be thought of as learning a probability distribution subject to the constraints specified by the labels. Training on *partially* labeled examples enforces constraints where the labels are known, while removing constraints where the labels are not known. During decoding, constraints may represent experimental knowledge about a particular protein; for example, the location of the N- or C-terminus, or the number of membrane-spanning segments. As an example of such a constrained HMM, a version of TMHMM was created explicitly to predict the topology of known 7-TM GPCRs [18]. More generally, these constraints can be “hard” (e.g., the N-terminal is known to be on the inside), or “soft” (e.g., there is conflicting experimental evidence, but it is likely that the N-terminal is on the inside).

One source of difficulty in defining an HMM for our task is related to the labeling of the training examples, both the uncertainty in the precise locations of the segment boundaries, and the occasionally missing (unknown) labels. Furthermore, there is a one-to-many association between the labels and the structural classes defined in the model, which typically subdivide many of the labeled regions, e.g., membrane, into two or more sub-regions with different emission distributions and/or duration models. Our DBN implementation allows for this one-to-many relationship between labels and states as well as the occasionally missing labels by expressing the relationship between the label and the state as a flexible constraint, including the use of a *wildcard* label which effectively removes the label-imposed local constraint on the state, while the probabilistic relationships (e.g., grammar constraints) between the state and the rest of the graph are maintained.

#### Virtual evidence

A flexible method for applying constraints on a DBN, while remaining within the graphical DBN framework, is to use a concept known as *virtual evidence* [15],[17],[19] (sometimes also referred to as *soft* evidence). The virtual evidence nodes typically represent binary random variables, and the evidence is that they are observed to be equal to 1. In this work we use two slightly different virtual evidence mechanisms. In the first, the virtual evidence node *c* is called an ‘observed child’ [19] and is used to induce a relationship between its (hidden and otherwise unconnected) parents. Consider, for example, three variables, *a*, *b*, and *c*, where *a* and *b* are the parents of *c*, and we observe that *c* = 1. We define Pr[*c* = 1|*a*,*b*] ∝ *f*(*a*,*b*) where *f*(*a*,*b*)≥0 can be used to express a preference for certain pairs (*a*,*b*), or forbid those for which *f*(*a*,*b*) = 0. Depending on the objectives, this relationship may be based on prior knowledge or it can be learned during training. In the second usage, the virtual evidence node *c* has a single parent *a* which we want to influence in some way. Again we observe *c* = 1, and set Pr[*c* = 1|*a*] = *f*(*a*) where *f*(*a*)≥0 expresses the desired influence. A further extension of this notion of virtual evidence, used during the decoding procedure (see Methods) allows position-dependent (i.e., time inhomogeneous) CPTs, i.e., Pr[*c_i_* = 1|*a*] ∝ *f*(*a*,*i*).

#### Duration modeling

DBNs also offer more flexibility in defining segment duration distributions. In a typical HMM, the duration associated with a state *s* follows a geometric distribution: Pr[*D_s_* = *d*] = *p*(1−*p*)*^d^* ^−1^, where *D_s_* is the random variable representing the duration of state *s*, *d* is a particular segment duration, and *p* is the probability of transitioning to a new state *q*≠*s*. If *p* = 1, then Pr[*D_s_* = 1] = 1. The geometric distribution is such that the single most likely duration is 1, the mean duration is 1/*p*, and any arbitrarily long duration can occur with non-zero probability. Although the geometric distribution is reasonable for some tasks, it is preferable in many applications to model an arbitrary but finite duration distribution (one with a hard limit on the maximum duration). In an HMM, this modeling is typically done using a ‘ladder’ or ‘chain’ of non-self-looping states, in which *D*_max_ distinct states are used to capture a finite duration distribution over [1,*D*_max_] by allowing certain states to be skipped with non-zero probability. This is the strategy adopted by the Phobius HMM [9]. Another common HMM strategy chains multiple geometric states together each with self-repeating loops, thus yielding a *negative-binomial* duration distribution [14]. A DBN can greatly simplify the specification and learning of a variety of complex duration behaviors within the DBN framework itself, without requiring large numbers of states and more complicated state-transition graphs. For example, the DBN presented in this work defines three basic duration behaviors, one of which will be associated with each state. One of these behaviors captures the geometric distribution described above. The other two are for finite-duration states: a fixed duration *D*, and a variable duration within a fixed window [*D*_min_,*D*_max_]. This latter case is expressed very easily by defining a duration distribution over a fixed range, and then sampling from this distribution to determine the actual segment duration. This duration modeling is similar to that implemented in the GHMM described by Kulp et al. [20], an early example of an extension to the basic HMM.

#### The Graphical Model Toolkit

In this work, we perform all training and inference in DBNs using the Graphical Model Toolkit [21] which includes generalized versions of the forward-backward, Baum-Welch, and Viterbi algorithms, and which supports all of the features mentioned above. For discrete variables, training consists of estimating the conditional probability table (CPT), Pr[*v*|*π_v_*], for each variable *v* given its parents *π_v_* such that the likelihood of the data is maximal. If all variables are observed during training, then estimating these tables is reduced to a simple counting task. If some variables are hidden, then the expectation-maximization (EM) algorithm [22] is used to find maximum likelihood estimates of the CPTs.

## Methods

### The Philius Model

**Figure 3:**
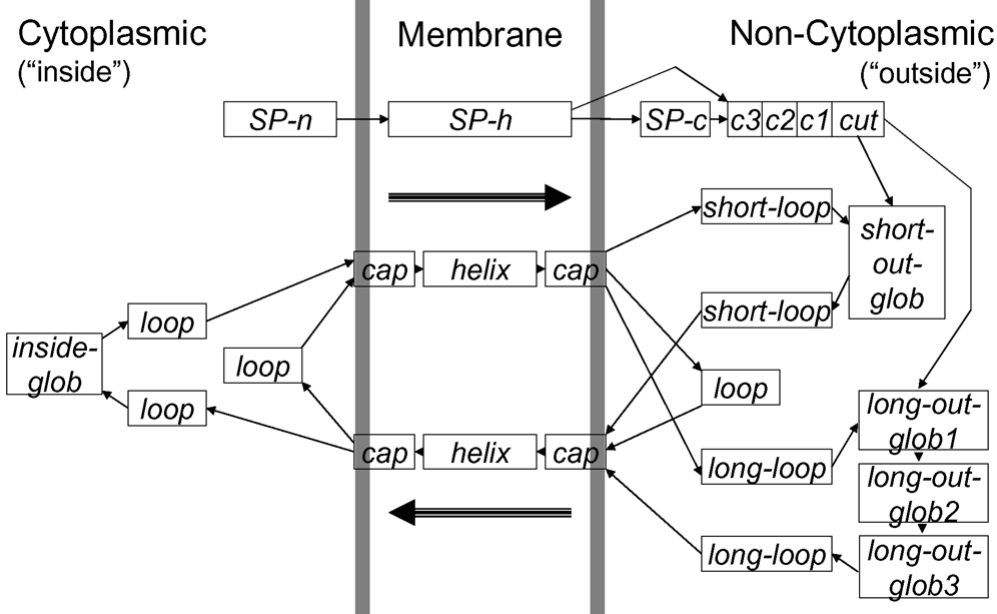
State transition diagram. Each rectangle represents a *state*, which is characterized by an emission distribution and a duration distribution. The state transition topology of Philius exactly mimics that of Phobius.

Philius's state transition diagram is shown in Figure 3. The model includes three basic regions–cytoplasmic, membrane, and non-cytoplasmic–each containing multiple states and representing one or more different topology labels. At this level of description, Philius exactly mimics Phobius. In the Phobius HMM, the states shown in Figure 3 are implemented as collections of HMM states, with transitions defined to produce the desired segment duration distributions. In Philius, by contrast, the duration modeling is explicit.

### Training

For the typical HMM as in Figure 1b, a state transition diagram along with the transition probabilities and emission distributions is sufficient to completely describe the model. The same DBN is used in training and decoding, the only difference being that the states are observed during (supervised) training and hidden during testing. With DBNs, it is common to use different graph topologies for training and decoding. Philius uses three different graphs, shown in Figure 2.

The training DBN shown in Figure 2a addresses the duration and labeling issues described earlier. The Markov chain backbone over the state nodes is the same as in a typical HMM, and the relationship between state *_i_* and state *_i_*_−1_ is defined by the usual state transition matrix, Pr[*s_i_*|*s_i_*_−1_], represented in the state transition diagram shown in Figure 3. Beyond the backbone, this DBN differs significantly from the standard HMM. Within each frame, the state node is related to three other random variables: the durationClass , the emissionClass, and the topoLabel. The first two are hidden variables, but in both cases the relationship to the state is a deterministic mapping that does not impact the computational complexity. The mapping from state to durationClass reflects which states share similar duration properties. Similarly, the mapping from state to emissionClass reflects which states share similar emission distributions. The emissionClass node is the one that ‘emits’ the amino acid according to the appropriate distribution. The amino acid is observed during training and during the first decoding stage.

The relationship between state and topoLabel is enforced using an *observed child* mechanism [19], i.e., the value of state is constrained by the observed value of topoLabel. There can be a many-to-one relationship between the state and the topoLabel: one value of topoLabel, such as *inside*, allows the state variable to take on several different values, while another label, such as *cleavage site* constrains the state variable to a single value. This approach is more flexible than the class-HMM described by Krogh in [23] in which each state emits a (class, observation) pair.

As previously described, the wildcard label places no restrictions on the current state, while the *sequence* of states remains constrained by the allowed state transitions and state durations, thereby preserving the grammar. Even with fully labeled training data, there is some uncertainty in the locations of the boundaries between adjacent segments. To account for this uncertainty and to allow the model more flexibility during training, we remove up to five labels on either side of every boundary (while keeping at least one label per segment), and replace these labels with the wildcard label. During training the model will adjust the location of the boundary in order to maximize the probability of each training example given the model parameters. Other researchers have addressed this issue with a two-stage training procedure in which an initial model is trained and then used to relabel the training data, before the final model is trained. This type of two-stage training approach may result in a final model that is overly dependent on the decisions made by the initial model. Our wildcard label approach allows us to train the model in a single pass, maintaining the expression of uncertainty regarding the labels, and can also be used in a semi-supervised setting, combining partially-labeled data with fully-labeled data.

The duration modeling for each duration class is handled by the stateCountDown and changeState nodes. Three basic types of duration models are allowed: (i) fixed and finite durations; (ii) random and finite durations; and (iii) geometric (possibly infinite) durations. The first two types are defined using a CPT Pr[*D* = *d*|*C_v_*], representing the probability that the duration of the current segment *D* is equal to *d*, conditioned on the duration class *C_v_*. The dimensions of this table are *D*_max_ by |*C_v_*|, where *D*_max_ is the maximum finite duration and |*C_v_*| is the number of different duration classes to be learned. When a transition to a *new* (different) state occurs, a randomly chosen duration is used to initialize the stateCountDown node. This value is decremented in each successive frame until it reaches a value of 1 whereupon the changeState node is set to true and a state transition is triggered in the next frame. The states with a geometric duration distribution are handled using a slightly different mechanism. For these states, the stateCountDown node is assigned the value of 0, which is not decremented in the subsequent frame. Instead, the binary changeState node is set randomly to true or false based on the self-looping probability *p* for the appropriate duration class.

The model is trained on labeled data (with wildcards as described above) using the EM algorithm. The free parameters learned during training consist of the start state probabilities, the transition probabilities for the few states that have more than one allowed next-state, the emission distributions for each emission class, the duration distributions for the finite duration classes, and the self-looping probabilities for the geometric duration classes, for a total of 388 free parameters. (There are 6 possible start-states, 4 states with more than one possible next state, 15 different emission classes, 87 finite-duration model parameters and 6 geometric-duration model parameters.) The emission class probabilities were smoothed by adding a single pseudo-count to each of the accumulated counts during training. Although the EM algorithm is only guaranteed to converge to a local maximum, in this case the uncertainties during training are only related to the exact placement of the segment boundaries and we found that repeated EM training runs did not result in significantly different parameters (data not shown).

### Decoding

The Viterbi algorithm is commonly used to find the most likely sequence of hidden states in an HMM given the observations and the model parameters. For a DBN, a generalized version of the Viterbi algorithm similarly finds the single most likely assignment to the set of all hidden variables **h** = [*h*_1_,…,*h_H_*] given the evidence variables **e** = [*e*_1_,…,*e_E_*] and the model parameters Θ:
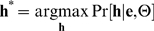



In this application, however, we are interested in finding the most likely sequence of labels λ^*^, where the variables in λ form a subset of **h**, but the best partial assignment λ^*^ is not necessarily contained in the best overall assignment h^*^. Computing λ^*^ is intractable in general [24], because it requires first that we compute the probabilities of all possible assignments and then sum over all assignments that correspond to each possible sequence of labels. In order to estimate the most likely sequence of labels, we have developed a novel two-stage approach. In the first stage, we compute the posterior probabilities for each λ by marginalizing out all other hidden variables. Defining a sequence of labels λ directly based on these posterior probabilities may produce a sequence that does not obey the grammar of the underlying model. Instead, we use the posterior probabilities on the labels in a second stage to influence the choice of the ‘best’ assignment h^*^, while enforcing the same grammar defined by the state transition matrix. Each of the two decoding stages uses a different graph than the one used in training, and these graphs are shown in Figure 3b and 3c.

This two-stage decoding is similar to the *posterior Viterbi* algorithm described in [25] and applied to predicting the topology of *β*-barrel membrane proteins, and is also similar to the *optimal accuracy decoding* used in [26] to combine information from homologous proteins. Both of these approaches use Viterbi-like algorithms to find the permissible sequence of states that maximizes some function of the posterior state probabilities. Here, we are effectively finding the permissible sequence of states that maximizes the product of the posterior label probabilities, subject to the topology grammar. By using DBNs combined with virtual evidence, there is no need to construct special-purpose inference algorithms; the only changes are in the definition of the topology of the graphical model and in the incorporation of the virtual evidence.

In the first stage decoding DBN, shown in Figure 2b, the observed topoLabel in the training graph is removed and replaced with a hidden topoState which is dependent on the current state and the previous topoState, and combines both the current topology label (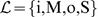) and whether or not the label has just changed (i.e., a new segment has been started). Incorporating this change-of-label information was found to significantly improve the precise localization of the signal peptide cleavage site. In addition, a new summary variable, pType , has been added which takes on one of four values in {G, SP+G, TM, SP+TM}, representing the four basic protein types. The pType node keeps track of whether or not a particular state assignment includes a signal peptide, and whether or not it includes a (non-SP) transmembrane segment. This is done by initializing pType = *G* and then or-ing together the pType from the previous frame with information from the current topoState to determine the pType up to and including the current frame. Full inference is performed on this graph to compute the posterior probabilities of all nodes given the evidence (the amino acid sequence) and the model parameters. Specifically, this first stage of the decoding produces as output the posterior probabilities for the topoState variable in each frame as well as the posterior probabilities for pType in the final (right-most) frame. Note that these posterior probabilities on the final protein type node should not be confused with a posterior probability on the location of the C-terminus of the protein; for each type in {G, SP+G, TM, SP+TM}, it represents the total probability, after all other hidden variables have been marginalized out, that the test protein is of that type.

The second stage decoding DBN, shown in Figure 2c, is significantly simpler than the other two graphs: the amino acid evidence has been removed along with the emissionClass node, as has the entire segment duration portion of the graph. In order to incorporate the information from the first stage, a new observed child node topo*_VE_* has been added in each frame. The parent of this new node is the topoState node, and the conditional relationship is defined, in a position- *in*homogeneous manner, based on the posterior label probability computed in the first stage:
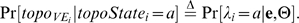
Because the posterior probabilities already include the effects of the transition, emission and duration probabilities, these no longer need to be included in the second stage. The output of the second stage of the decoder is the topology resulting from the Viterbi assignment to the hidden variables in Figure 2c. The Viterbi topology λ^v^ is now much closer to the optimal solution λ^*^ because of the inclusion of the posterior probabilities from the first stage.

Experimental information can also be easily incorporated into this decoding process. For example, if the protein type is known, then the final *pType* node can be constrained to match. If other information is known, such as the location of the C-terminus or details regarding particular membrane-spanning segments, this too can be easily incorporated as additional evidence constraining the topoState nodes in those frames where the evidence exists.

### Confidence Scores

In the Results section, we describe three types of confidence scores: protein type, per-segment, and topology. The first score reflects Philius's confidence in the assignment of the protein type–G, SP+G, TM or SP+TM. The protein type score is computed using the posterior probabilities for the pType variable in the final frame of the first stage decoding DBN. This computation produces a single set of probabilities Pr[*y*] for each evaluated protein. The second stage of the decoder produces the topology prediction and the predicted protein type *yˆ*. The confidence score associated with the protein type prediction is the posterior probability Pr[*yˆ*]. The second type of score is the per-segment score, which represents an estimate of the accuracy of the label and boundaries of a particular segment. For this score, we use the Viterbi segmentation from the second stage and compute the arithmetic mean of the first stage posterior probabilities within that segment for the Viterbi-assigned topology label. The third score applies only to transmembrane proteins and reflects Philius's confidence in the overall predicted topology. We define this score as the minimum segment score over all predicted membrane segments and the N-terminal and C-terminal segments.

### Datasets

We used the Phobius dataset [9] during model development. This dataset consists of four non-overlapping subsets of 1087 globular (G) proteins, 1275 globular proteins with signal peptides (SP+G), 247 transmembrane (TM) proteins and 45 transmembrane proteins with signal peptides (SP+TM). The maximum homology among the 247 TM proteins is 80%, and the maximum homology among the 45 SP+TM proteins is 35%. The same cross-validation folds and the same labels that were used to train and test Phobius were also used in this work.

Two additional datasets were obtained and used in the final testing and evaluation of the model: the SCAMPI dataset [27] of 124 transmembrane proteins (http://octopus.cbr.su.se/index.php?about=download) and the SignalP 3.0 [28] training dataset. The labels in the SCAMPI dataset include re-entrant regions which do not completely span the membrane. These were removed and relabeled as *inside* or *outside* because Philius does not currently model those types of segments. The maximum homology among these 124 proteins is 40%. Based on homology between these and the original Phobius TM proteins, this set was divided into one set of 77 proteins that does not overlap the Phobius dataset (maximum homology 80%), and one set of 47 proteins that does. For the purposes of training and testing Philius we only used the signal peptide portion of the SignalP dataset, combining the eukaryotic and bacterial proteins into a single set of 1728 proteins. Truncated versions of these proteins were used in training because the labels covered only the signal peptide and cleavage-site of each protein.

## Results

We evaluated the performance of Philius on the development dataset using ten-fold cross-validation. We measured the performance of the model as well as the accuracy of all three types of confidence scores. For proteins containing a signal peptide, we also considered the accuracy with which the cleavage site is localized.

We chose to compare our method to Phobius because it is the only method that we know of that simultaneously predicts signal peptides and complete transmembrane topologies. Several methods, such as MemBrain [29] and Proteus [30], predict transmembrane helices and signal peptides, but without any topological (inside/outside) information. The web server PONGO [31] gives predictions from individual transmembrane topology and signal peptide predictors without combining the individual predictors.

### Protein Type Classification

Initially, we evaluate how accurately Philius identifies a given protein's class as G, SP+G, TM or SP+TM. Table 1 shows the performance of Phobius and Philius at this task using accuracy, precision, sensitivity, specificity and Matthews correlation coefficient as metrics. Note that, because the SP+TM subset consists of only 45 examples, fewer than 2% of the 2654 proteins in the development set, we will sometimes group them together with the other TM proteins to provide more meaningful statistics. The largest difference between Philius and Phobius at this level is in the precision for the TM and SP+TM category, for which Philius calls 29% fewer false positives than Phobius. (Phobius finds 265 of the 292 true positives, and miscalls 82 of the 2362 true negatives; on the same data, Philius finds 268 TPs and miscalls 58 TNs.) Overall, the performance on the G and SP+G subsets has decreased slightly in exchange for an improvement on the TM subset which is of greatest interest. Note that the class sizes in this dataset are skewed (48% SP+G, 41% G, and 11% TM and SP+TM), and that compared to a complete proteome, the transmembrane proteins are underrepresented in this dataset by a factor of 2 to 3.

**Table 1:** Phobius and Philius protein type classification performance on the development set: for each protein class, the fraction of the dataset of that type, and the accuracy, precision, sensitivity, specificity, and Matthews correlation coefficient.

		Accuracy		Precision		Sensitivity		Specificity		Matthews C	
Protein Type	Data %	Phobius	Philius	Phobius	Philius	Phobius	Philius	Phobius	Philius	Phobius	Philius
TM, SP+TM	11%	0.98	0.98	0.79	0.87	0.91	0.92	0.98	0.98	0.83	0.88
SP+G	48%	0.96	0.95	0.97	0.95	0.94	0.95	0.97	0.96	0.92	0.91
G	41%	0.97	0.97	0.97	0.97	0.96	0.95	0.98	0.98	0.94	0.93

**Figure 4:**
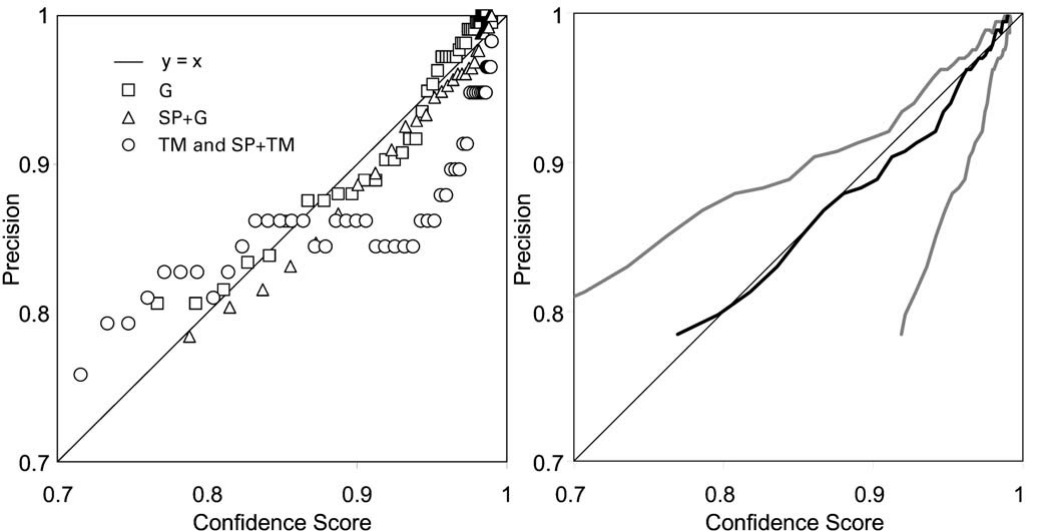
Protein-type classification precision vs confidence score computed by sorting the proteins by score and computing the average score and precision within a sliding window. Left: precision vs average score for each of the three main protein types. Right: average (black) and average ±one standard deviation (gray) across all proteins.

For each prediction, Philius reports a protein type confidence score, and Figure 4 shows that this score correlates extremely well with the precision of the classification decision. Furthermore, on this dataset, more than 70% of the confidence scores are greater than 0.95. For the TM and SP+TM proteins (the smallest class), the confidence score tends to be somewhat optimistic, as indicated by the points below *y* = *x*. We attribute this skew to the fact that the model was tuned to maximize the balanced accuracy across the three major classes.

### Segment-Level Prediction

Next, we evaluated the performance of Philius at the segment level. Philius predicts four basic segment types: signal peptide, transmembrane segment, and inside and outside loops. For a transmembrane segment, the predicted segment must overlap the annotated segment by at least five amino acids to be deemed correctly identified. In order to correctly identify a signal peptide, the model must only predict its existence at the N-terminus of the protein. Because many of the inside and outside loops are very short, the overlap required for these segments is only one amino acid. The sensitivity and precision of the model in predicting each of these segment types is shown in Table 2. Accuracy and specificity cannot be calculated at the segment level, because there is no sensible way to define the number of true negatives. Results for outside segments are reported for all segments as well as for the subset of outside loops within transmembrane proteins (i.e., those with at least one non-SP TM segment). All of the inside segments reported are loops within TM proteins. Predicting whether a loop between two adjacent TM segments is on the ‘inside’ or on the ‘outside’ of the membrane is clearly the most challenging aspect of this task.

**Table 2:** Segment-level metrics.

Segment Type	Sensitivity	Precision
SP	0.96	0.96
TM	0.94	0.92
Inside	0.87	0.85
Outside(TM)	0.89	0.88
Outside(all)	0.97	0.97

**Figure 5:**
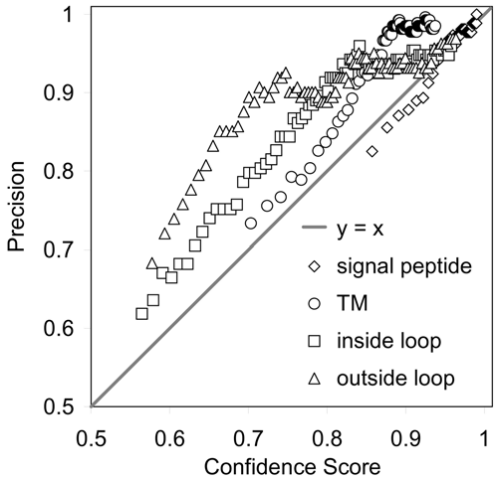
Segment-level classification precision vs score for each of the segment types (excluding the ‘outside’ segments of G and SP+G proteins).

As shown in Figure 5, the segment-level scores correlate well with precision. The membrane segment and inner and outer loop scores tend to be conservative, as indicated by the points above *y* = *x*. The segment score should be interpreted conditioned on the assumption that the protein type has been correctly predicted.

### Signal Peptide Cleavage Site Accuracy

Although the precise boundaries of the membrane segments of a transmembrane protein are somewhat difficult to define, the cleavage site of a signal peptide can be very precisely defined if the first amino acid of the mature protein is known. We therefore also evaluated Philius' ability to correctly localize the signal peptide cleavage site.

Combining the SP+G and the SP+TM proteins into one group and the G and TM proteins into another, the development dataset contains 1320 proteins with signal peptides and 1334 without. In the cross-validation experiment, Philius predicts 1271 true positives, 1278 true negatives, 49 false negatives, and 56 false positives (accuracy = 0.96, precision = 0.96, sensitivity = 0.96, and specificity = 0.96).

Of the 1271 predicted true positives, in 948 cases (75% of the predicted positives, and 72% of all positives), the annotated cleavage site is found exactly. Among the errors, there is very little skew in the localization error: in 51% of the cases, the cleavage site is predicted “early” (median offset is 3 amino acids), and in 49% of the cases the cleavage site is predicted “late” (median offset is 2 amino acids).

### Full Topology Prediction

For proteins with transmembrane segments (with or without a signal peptide), it is important to be able to correctly predict the entire protein topology. Getting this prediction right requires not only that all of the transmembrane segments be correctly identified, but that the loop regions between the membrane segments be correctly localized. Grouping the TM and SP+TM proteins together, Philius predicts the correct topology for a total of 212 out of 292 proteins (72.6%). For comparison, Phobius predicts 198 correct topologies (67.8%) on this same dataset. Table 3 shows the confusion matrices for Philius and Phobius. Within each half of the table, values on the diagonal represent correct protein-type predictions, while off-diagonal values represent errors. For G and SP+G proteins, a correct protein-type prediction implies a correct topology, whereas for TM and SP+TM proteins this is not necessarily the case. For these proteins, the first number represents the number of correct complete topologies while the second number represents the number of incorrect topologies. (Incorrect protein-type calls necessarily imply incorrect topologies.)

**Table 3:** Confusion matrices for Phobius and Philius.

Phobius	G	SP+G	TM	SP+TM	Philius	G	SP+G	TM	SP+TM
G	1042	25	20	0	G	1033	43	11	0
SP+G	27	1207	20	21	SP+G	25	1200	19	31
TM	5	9	157/66	10	TM	8	9	172/54	4
SP+TM	0	1	2	41/1	SP+TM	0	1	2	40/2

Rows are true protein types, and columns are predicted protein types. Where there are two numbers, the first number represents the number of proteins for which the full topology was correctly predicted, while the second number represents the number of proteins for which the protein type was correct but the full topology was not. These results are from the development dataset.

**Figure 6:**
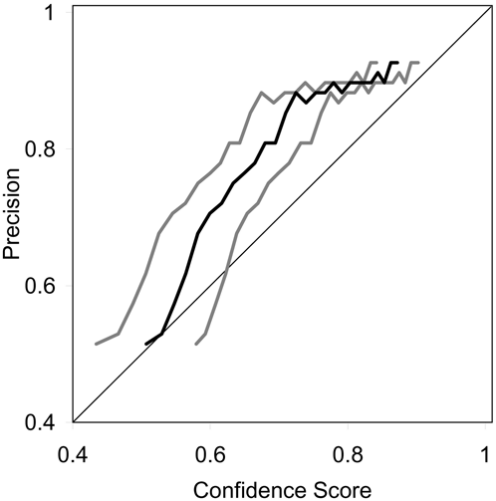
Full-topology prediction precision vs score for the TM proteins. The black line is the average score within the sliding window used to estimate the precision, and the gray lines indicate the average plus and minus one standard deviation.

Figure 6 shows that the full-topology confidence score correlates reasonably well with the observed precision for the transmembrane proteins in the dataset. As with the segment scores, the full-topology confidence score should be interpreted conditioned on the assumption that the protein type has been correctly inferred.

### Results on Test Data

**Figure 7:**
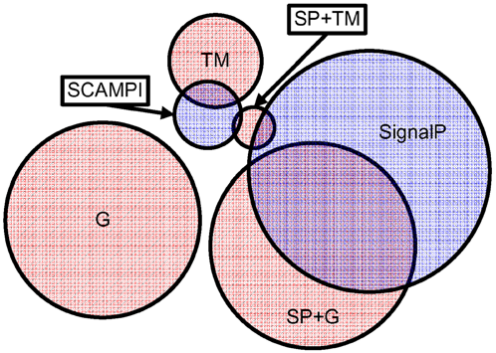
Original Phobius datasets (G, SP+G, TM and SP+TM) and new SignalP and SCAMPI datasets. Figure is approximately to scale.

Following the model-development phase, we evaluated Philius on an enhanced dataset that includes the SCAMPI dataset [27] and the SignalP 3.0 dataset of signal peptide proteins [27]. These new datasets partially overlap the original Phobius datasets that were used during model development as shown in Figure 7. We incorporated this new data to create a new set which we used for a final round of ten-fold cross-validated training and testing. This new dataset was made up of the original Phobius G and SP+TM subsets, the SignalP signal peptide set (combining eukaryotic and bacterial proteins), and a merged TM set created by combining the 124 TM proteins from the SCAMPI set with the 200 non-homologous TM proteins from the Phobius TM subset, for a total of 324 TM proteins.

Results were evaluated in two areas: full-topology accuracy on the transmembrane proteins, and signal peptide prediction accuracy on the SignalP dataset. The full-topology accuracy on the TM proteins after performing ten-fold cross-validation on this new dataset is summarized in Table 4. The accuracies reported in the first 2 rows of the table are consistent with one another and with the accuracy of 72.6% reported on the development set. Comparing the last two rows in the table it is clear that the novel portion of the SCAMPI dataset contains membrane proteins that are more difficult to predict. This is likely due to the presence in the SCAMPI set of 20 proteins known to have one or more re-entrant segments. Of these 20 proteins, all but one are in the SCAMPI \ Phobius set, and the full-topology accuracy on these 19 proteins is only 53% (10/19).

**Table 4:** Philius full-topology accuracy on new merged TM dataset (top row).

TM Dataset	Size	Correct Count	Correct %
Phobius ∪ SCAMPI	324	230	71.0%
SCAMPI only	124	90	72.6%
Phobius ∩ SCAMPI	47	37	78.7%
SCAMPI \ Phobius	77	53	68.8%

The accuracy on various subsets of the merged set are listed below.

Training and testing Phobius in the same way on this new merged dataset yielded an overall TM topology accuracy of 62.7% (203 out of 324). Compared to Phobius, on this new dataset, Philius achieves a relative increase of 13% in the number of correct topologies (230 correct topologies vs 203).

The signal peptide performance is improved over that reported for the development dataset. We attribute this improvement to the higher quality SignalP dataset. On 1728 signal peptides, Philius predicted 1679 true positives and 49 false negatives (30 were classified as transmembrane proteins, while 19 were classified as globular proteins) for a sensitivity of 0.97 (compared to 0.96 on the Phobius SP set). Furthermore, 1292 cleavage sites are predicted exactly, representing 75% of all signal peptides in the test set, compared to 72% when trained and evaluated on the Phobius SP set.

Although we combined the eukaryotic and bacterial signal peptides during training, we also report in Table 5 the results broken down by taxon. For these results, the positive set is the SignalP dataset of signal peptides (with the counts for each subset as shown in the table), and the negative set is the Phobius globular protein set (1087 proteins). The results represent the summary from a ten-fold cross-validation experiment. Although we are not using the same set of negative (non-SP) proteins and thus cannot exactly replicate the experiments leading to the SignalP 3.0 performance figures reported by Bendtsen et al. in [28], Philius' detection and discrimination of signal peptides is comparable to that reported for SignalP-HMM for eukaryotes and Gram negative bacteria. The cleavage site accuracy reported here for Philius is slightly worse than SignalP-HMM for the eukaryote and the Gram negative sets (down 4% and less than 3% respectively), but is significantly worse for the Gram positive set (down 24%). This decline in performance is to be expected, considering that we trained a single model for all three categories, and the Gram positive signal peptides are significantly different from the other two types.

**Table 5:** Philius signal peptide discrimination (accuracy, precision, sensitivity, specificity, and Matthews correlations coefficient) and cleavage-site accuracy (fraction of all SPs detected for which the cleavage-site was predicted exactly).

Dataset	Count	Acc	Prec	Sens	Spec	cc	C-Site
Eukaryotes	1192	0.97	0.97	0.97	0.97	0.94	72.4%
Gram−	370	0.97	0.91	0.98	0.97	0.92	87.8%
Gram+	167	0.97	0.81	0.96	0.97	0.86	62.3%
All	1729	0.97	0.98	0.97	0.97	0.94	74.7%

The negative set contained 1087 globular proteins.

The key difference between Philius and SignalP, however, is that SignalP is trained to discriminate between proteins with and without signal peptides, excluding transmembrane proteins, whereas Philius has been trained to discriminate between proteins with and without signal peptides *and* those with and without other (non-SP) membrane-spanning segments. It has previously been reported that SignalP 3.0 falsely predicts 21% (52 of 247) of the Phobius TM dataset as containing signal peptides and that 30–65% of all predictions from SignalP 3.0 on whole proteomes overlap with TMHMM 2.0 predictions [8]. Philius, in contrast, predicts only 5% (13 of 247) of the Phobius TM dataset as containing a signal peptide.

### *S. cerevisiae* Membrane Proteome

Kim et al. [32] described the experimental localization of the C-terminus for 617 *Saccharomyces cerevisiae* proteins predicted by TMHMM to be multi-spanning membrane proteins using a reporter construct. Based on consistent experimental results as well as BLAST homology searches, the C-terminal location could be confidently assigned for a total of 546 proteins. For 69% of the 546 proteins, the initial TMHMM prediction of the C-terminal location agreed with the experimental result. New topology predictions were made using both TMHMM and prodiv-TMHMM [33] constrained by the experimentally determined C-terminal location.

**Figure 8:**
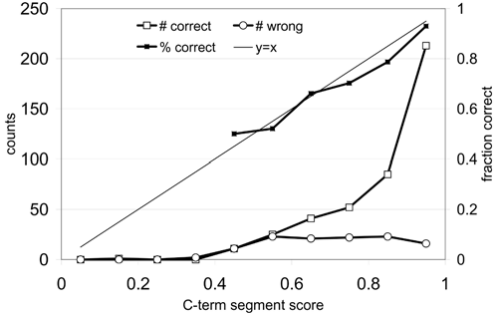
The total counts and fraction of correct C-terminal localizations as a function of C-terminal segment confidence score for 546 yeast proteins with experimentally assigned C-terminal locations.

The Philius predictions for the 546 proteins described above match the experimentally assigned C-terminal location 78% of the time (428 out of 546). For those C-terminal segments that were correctly predicted by Philius, the median confidence score was 0.90. For those incorrectly predicted, the median score was 0.72. Figure 8 shows the total counts and fraction of correctly localized C-terminals as a function of the C-terminal segment confidence score.

Constrained Philius topology predictions were then made and compared to those given in [32]. The Philius-predicted topology matched both TMHMM and prodiv-TMHMM for 41% of the 536. (For 10 out of the original 546 proteins, the length of the protein given in the supplementary data of [32] did not match the length of the ORF of the same name in the YRC database, so these proteins were disregarded for all other comparisons.) proteins, only prodiv-TMHMM for 21%, only TMHMM for 16%, and neither for 22%. The constrained predictions from TMHMM and prodiv-TMHMM match each other for 48% of the 546 proteins.

The constrained Philius predictions included 40 topologies containing a predicted N-terminal signal peptide. Of these, 31 signal peptides had high confidence scores (greater than 0.9), and all but one of these were also classified as containing a signal peptide by SignalP 3.0. Of these 30 putative signal peptides identified both by Philius and by SignalP, TMHMM annotates 18 (60%) as transmembrane segments. Four of these proteins are classified as SP+G by Philius, indicating that the mature protein is likely a globular protein and not a membrane protein. Of these proteins, three (YFL051C, YNL019C, and YNL033W) are putative proteins, and the fourth (YFL067W) is of uncharacterized function.

### Predictions on YRC Database

A final version of Philius, trained on all of the training data, was used to predict and score the protein type and topology for all 6.3 million proteins in the YRC public data repository [11] as of March 24, 2008. This database contains Uniprot/SwissProt, the NCBI non-redundant database, the MIPS protein sequence database, and a variety of organism-specific databases, including the *Saccharomyces* Genome Database, Sanger's *S. pombe* database (pompep), Wormbase, Flybase and The *Arabidopsis* Information Resource. Running Philius on this set required approximately 7.2 s per protein, for a total of approximately 1.5 years of CPU time.

A summary of the predictions can be found in Table 6. The median protein type confidence scores are very high for all protein types. The median topology confidence score for TM proteins is 0.69, which agrees with the typical topology accuracy of 70%. Table 7 shows the relative representation of the four basic protein types, for four species. The total fraction of predicted membrane proteins, between 22% and 29% is consistent with previous estimates. Table 8 shows the fraction of predicted TM and SP+TM proteins that have a single membrane-spanning segment in the mature protein. Single-spanning membrane proteins represent approximately 20% to 35% of all membrane proteins, and an even larger fraction of membrane proteins with signal peptides. For putative multi-spanning transmembrane proteins, proteins predicted to contain an even number of membrane segments outnumber those predicted to have an odd number of membrane segments nearly 2 to 1 (data not shown). This enrichment of membrane proteins with an even number of TM segments may be due to internal duplication events resulting in an even number of TM segments, or the process of membrane insertion may be optimized for pairs of segments. Although the N-terminus of a membrane protein is in general more likely to be on the cytoplasmic side of the membrane, this bias is strongest for proteins with an even number of membrane segments. Two extreme examples illustrate this phenomenon: less than 41% of the putative seven-transmembrane segment proteins are predicted to have the N-terminal on the inside (the large family of GPCR proteins have the N-terminal on the outside), whereas 96% of the proteins predicted to have twelve transmembrane segments are predicted to have the N-terminal on the inside. This same phenomenon was seen in our training data and in other genome-wide prediction studies [32],[34].

**Table 6:** Overall YRC predictions on 6.3 million proteins: number and relative fraction of each protein type, median protein type confidence score, and median TM topology confidence score (when applicable).

Protein Type	Count	Percentage	Median Type Confidence	Median Topology Confidence
G	4,248,628	67.1%	0.98	
TM	1,280,117	20.2%	0.99	0.69
SP+G	698,534	11.0%	0.97	
SP+TM	101,224	1.6%	0.91	0.78

**Table 7:** This table shows, for a few different organisms, the total number of proteins for which predictions were made and the relative fractions of the four basic protein types.

Organism	Count	G	SP+G	TM	SP+TM
*E. coli*	4,929	61%	17%	21%	1%
*S. cerevisiae*	6,633	70%	7%	21%	1%
*C. elegans*	22,969	55%	15%	26%	3%
*H. sapiens* (HUPO)	16,941	60%	15%	18%	7%

**Table 8:** This table shows the fraction of predicted TM and SP+TM that have a single membrane-spanning segment in the mature protein.

Organism	Total TM	ss	Total SP+TM	ss
*E. coli*	1,032	19%	35	49%
*S. cerevisiae*	1,416	35%	99	68%
*C. elegans*	5,919	29%	778	67%
*H. sapiens* (HUPO)	3,042	26%	1,222	74%

**Figure 9:**
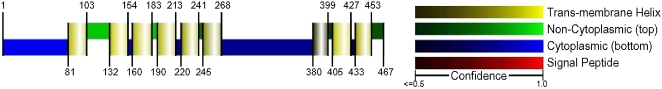
Philius topology prediction for the human presenilin protein as shown on the YRC web-page. The diagram shows the nine membrane-spanning regions as vertical cylinders, and the cytoplasmic and non-cytoplasmic segments as horizontal bars. Each segment is colored according to type and shaded according to the confidence score. The seventh membrane-helix is missed by many topology predictors and is assigned a relatively low confidence score by Philius and as such is shaded gray. Because of this one low-confidence membrane segment, the location of the C-terminus is less confidently assigned than the location of the N-terminus. On the YRC web page, this diagram is accompanied by the type confidence and topology confidence, as well as a copy of the protein sequence, color coded by segment type. Placing the mouse over any part of the topology diagram or the color-coded sequence will produce a pop-up showing the segment type, confidence, and boundary locations.

Figure 9 shows the Philius topology prediction for the human presenilin protein. This topology matches the nine-transmembrane topology which has been recently described [35],[36] and is supported by experimental evidence. The nine membrane-spanning regions are shown as vertical cylinders and the cytoplasmic and non-cytoplasmic segments as horizontal bars. Each segment is colored according to type and shaded according to the confidence score. The seventh membrane-helix is missed by many topology predictors and is assigned a relatively low confidence score by Philius and as such is shaded gray. The protein type score for this protein is 0.99, and the full-topology score is 0.56.

## Discussion

We have described Philius, a DBN-based approach to transmembrane protein topology prediction. Philius incorporates a two-stage decoding procedure that approximates the most likely label sequence given the protein sequence, a flexible way of handling uncertainty in training labels or partially labeled data, three different types of duration models, and a simple mechanism for tying parameters in order to limit model complexity. We have shown improvements in topology prediction accuracy over Phobius and comparable signal-peptide discrimination to SignalP-HMM. Furthermore, Philius uses a probabilistic framework to derive three informative confidence measures which have been shown to correlate well with observed precision. Finally, we have made available through the YRC web page a prediction server and 6.3 million predicted protein topologies. The predictions provide a global view of membrane protein topology and are a significant resource for scientists interested in understanding protein structure and function.

With respect to the transmembrane protein topology prediction task, we plan to improve Philius in several respects. First, it has previously been shown that the performance of Phobius could be increased from 67.8% to 74.7% correctly predicted TM topologies by including homologs during the decoding stage [26]. Philius currently achieves 72.6% accuracy on the same dataset. We believe that Philius's performance could be similarly improved by exploiting homologs. Other directions for future work include learning periodic motifs (such as the hydrophobic moment [37]) in transmembrane helices, and including parallel tracks of information, such as hydrophobicity measures, in addition to the amino acid sequence. A model that differentiates between single-spanning and multi-spanning membrane proteins may also better capture some of the diversity among these proteins, at the risk of data-sparsity problems. However, including additional features such as hydrophobicity or otherwise clustering the amino acids may help to limit over-fitting to the training data. Furthermore, most existing membrane protein models, including Philius, are guilty of over-simplifying the problem, ignoring, for example, re-entrant segments which penetrate but do not completely span the membrane, or interfacial helices which run roughly parallel to the membrane surface [38]. Modeling and predicting these types of features without reducing the accuracy on more “conventional” membrane proteins remains an open problem.

Recently, some insight has been gained into which properties of a protein govern the insertion of its membrane segments. Specifically, it has been shown that for a potential transmembrane helix of a given protein, the apparent free energy of insertion Δ*G*_app_ of a TM helix can be expressed as a function of the concentration ratio *K*_app_ between the membrane integrated and the non-integrated forms: Δ*G*_app_ = −*RT* ln *K*_app_ [39],[40]. Furthermore, this Δ*G*_app_ can be approximated as a sum of position- and residue-dependent contributions from each amino acid in the helix, plus a hydrophobic moment contribution and a length correction [27],[40]. The additive nature of Δ*G*_app_, neglecting the hydrophobic moment term, supports the conclusion that probabilistic models in which the probabilities of individual amino acids are multiplied together, or equivalently the log-probabilities are summed, provide an accurate representation of the underlying membrane integration process. The length correction term can be compared to log Pr[*D_h_*], where *D_h_* is the length of the core membrane helix and Pr[*D_h_*] is learned. Within the DBN framework, it is also possible to incorporate additional dependencies between nearby amino acids in order to capture effects such as the hydrophobic moment.

Since their introduction to biological sequence analysis [41], hidden Markov models have been considered one of the best ways to model amino acid and DNA sequences. DBNs generalize HMMs and offer a number of significant advantages. While adding complexity to an HMM requires an ever-expanding state space, a DBN can be used to more precisely describe the relationships desired among the random variables, thereby limiting the complexity only to what is actually needed. Because DBNs expose additional factorizations that might not be apparent in an HMM, DBNs may require fewer parameters and allow computationally more efficient probabilistic inference procedures than the corresponding HMM. Recently, Yao et al. [42] have applied DBNs to the task of secondary structure prediction and it seems like a logical step to similarly extend other applications such as gene prediction [43], protein homology detection [23], and coiled-coil prediction [44] from HMMs to DBNs. The DBN used here for protein topology prediction can easily serve as the basis for any similar segmentation and labeling task simply by specifying a different set of states and a different grammar.
